# Berries and Human Health: Mechanisms and Evidence

**DOI:** 10.3390/nu15112527

**Published:** 2023-05-29

**Authors:** Daniela Martini, Mirko Marino, Cristian Del Bo’

**Affiliations:** Department of Food, Environmental and Nutritional Sciences, Università degli Studi di Milano, 20133 Milan, Italy; daniela.martini@unimi.it (D.M.);

Berry fruits (e.g., blueberry, cranberry, strawberry, raspberry, blackcurrant) contain a wide range of nutrients such as fiber, vitamins, minerals, and bioactive compounds such as (poly)phenols. Emerging scientific evidence supports their health-promoting potential against oxidative stress, inflammation, vascular dysfunction, and numerous metabolic dysregulations. However, most of the evidence derives from in vitro and animal models, while observations from human studies deserve further investigation. The Special Issue “Berries and Human Health: Mechanisms and Evidence” was open for submission of original research manuscripts focused on (i) dietary intervention studies exploring the role of berries and their (poly)phenols in the protection and promotion of human health; (ii) cell culture and animal studies devoted to evaluating the molecular mechanisms underpinning the modulation of metabolic and functional activities; (iii) systematic reviews and/or meta-analyses investigating the impact of berries in the modulation of risk factors and health outcome.

This Special Issue features six contributions: two original articles [1,2] and four reviews [3,4,5,6].

The two articles focused on the anti-inflammatory effect of two different berries in animal models [1,2]. In detail, Pemmari and colleagues [1] investigated how supplementation with cloudberry (*Rubus chamaemorus* L.) attenuated the development of metabolic inflammation in a high-fat diet mouse model of obesity. Results showed that 6- and 12-week supplementation with cloudberry prevented the rise in the systemic inflammation marker serum amyloid A and the hepatic inflammation/injury marker alanine aminotransferase, as well as the increase in the expression of many inflammation-related genes in the liver and adipose tissue.

Kim and coworkers [2] evaluated the effect of a treatment (10 weeks) with blackcurrant in mice with type 2 diabetes (T2DM). The authors found that the intervention significantly improved the homeostatic model evaluation of glucose, insulin, and insulin resistance (HOMA-IR) indices, diabetic blood markers, cardiac function markers and cardiac thickening, and elevated levels of inflammatory cytokines in cardiac tissue of T2DM mice. Overall, these results suggest that blackcurrant consumption may play a role in ameliorating diabetes-induced cardiovascular complications in mice with T2DM.

The four reviews collected in the Special Issue cover different topics. The review by De Amicis and colleagues [3] focuses on the effect of berries on cognitive function in healthy people. The authors collected findings from 12 studies that included almost 400 participants. Positive outcomes of berry consumption (mainly blueberry and hawberry) were found for all cognitive domains and, in most cases, they were demonstrated in more than one study and using different methodologies, thus supporting the consumption of berries in healthy populations to prevent cognitive decline.

The remaining three reviews focus on the effects of berries on markers of cardio-metabolic health. Vendrame et al. [4] provided an overview of the evidence for the role of berries on blood pressure and hypertension. Results showed that, despite there being insufficient evidence to support the existence of a direct blood-pressure-lowering effect, there is stronger evidence that chokeberries act indirectly through the improvement of the endothelial function to normalize blood pressure in subjects that are already hypertensive. Mixed results were found for cherries, blackcurrants, blueberries, and raspberries. These findings were also corroborated by the revision of Olecho et al. [5], who summarized chokeberry’s effects on various metabolic parameters derived from 16 studies published from 2000 to 2021. Overall, the authors found that chokeberries might have a positive effect against hypertension besides improving dyslipidemia and increasing the body’s antioxidant defense mechanisms.

Finally, the review by Venturi and coworkers [6] summarized the available evidence derived from 17 human intervention trials related to the effect of berries in the modulation of metabolic syndrome. Results showed positive effects mostly related to lipid profile and inflammation (e.g., reduction in interleukin-6 and tumor necrosis factor-alpha). At the same time, conflicting findings were documented for anthropometric parameters, blood pressure, and fasting blood glucose levels.

Taken together, results from the papers included in this Special Issue provide further evidence about the potential beneficial effects of berries on markers of human health, mostly related to cardiometabolic health and cognitive function. However, due to the heterogeneity of results found within studies, further evidence is needed to better elucidate the valuable role of the inclusion of berry fruits in the diet and the mechanisms that underpin such potential health effects.
